# A mindfulness-based, cognitive, social, digital relapse-prevention intervention for youth with depression in Australia: study protocol for a randomised controlled trial of Rebound

**DOI:** 10.1136/bmjopen-2024-088695

**Published:** 2024-11-27

**Authors:** Shaminka N Mangelsdorf, Daniela Cagliarini, Yong Yi Lee, Cathrine Mihalopoulos, Virginia Liu, Lee Valentine, Sarah Bendall, Peter Koval, Simon D'Alfonso, Christopher Davey, Penni Russon, Jess Phillips, Cesar Gonzalez-Blanch, Brendan Pawsey, Richard M Ryan, Alexandra Parker, Sarah Hetrick, Simon Rice, Reeva Lederman, Helen Herrman, Greg Murray, John Gleeson, Mario Alvarez-Jimenez

**Affiliations:** 1 Orygen, Parkville, Victoria, Australia; 2 Centre for Youth Mental Health, The University of Melbourne, Parkville, Victoria, Australia; 3 Monash University Health Economics Group, School of Public Health and Preventive Medicine, Monash University, Melbourne, Victoria, Australia; 4 Queensland Centre for Mental Health Research, Wacol, Queensland, Australia; 5 School of Public Health, The University of Queensland, Herston, Queensland, Australia; 6 Melbourne School of Psychological Sciences, The University of Melbourne, Parkville, Victoria, Australia; 7 Research Group of Quantitative Psychology and Individual Differences, KU Leuven, Leuven, Belgium; 8 School of Computing and Information Systems, The University of Melbourne, Parkville, Victoria, Australia; 9 Department of Psychiatry, The University of Melbourne, Parkville, Victoria, Australia; 10 School of Languages, Literatures, Cultures and Linguistics, Monash University, Melbourne, Victoria, Australia; 11 School of Psychological Sciences, Monash University, Melbourne, Victoria, Australia; 12 University Hospital Marques de Valdecilla-IDIVAL, Santander, Spain; 13 Mercy Health, Melbourne, Victoria, Australia; 14 Institute for Positive Psychology and Education, Australian Catholic University, North Sydney, New South Wales, Australia; 15 College of Education, Ewha Womans University, Seoul, South Korea; 16 Institute for Health and Sport, Victoria University, Melbourne, Victoria, Australia; 17 Department of Psychological Medicine, The University of Auckland, Auckland, New Zealand; 18 Movember Men’s Health Institute, Melbourne, Victoria, Australia; 19 Centre for Mental Health and Brain Sciences, Swinburne University of Technology, Hawthorn, Victoria, Australia; 20 Healthy Brain and Mind Research Centre, School of Behavioural and Health Sciences, Australian Catholic University, Fitzroy, Victoria, Australia

**Keywords:** Randomized Controlled Trial, Psychosocial Intervention, Depression & mood disorders

## Abstract

**Introduction:**

Major depressive disorder (MDD) causes significant disease burden and functional impairment during adolescence and young adulthood. While most young people recover from their first episode, around two-thirds will experience one or more relapses, which can become more severe and treatment-resistant with each episode. To address relapse in MDD, we developed a moderated online social therapy platform (titled *Rebound*) that integrates: (i) peer-to-peer social networking; (ii) tailored third-wave therapeutic content targeting mindfulness, self-compassion and rumination; and (iii) three types of human support (clinicians, peer workers, career consultants), informed by self-determination theory. The aim of this trial is to determine whether, in addition to treatment as usual (TAU), *Rebound*, an 18-month complex digital intervention, is superior to 18 months of enhanced TAU in preventing relapse and managing depressive symptoms.

**Methods and analysis:**

This study is a rater-masked randomised controlled trial. The treatment conditions include *Rebound* plus TAU or enhanced TAU alone. We aim to recruit 255 young people with at least one episode of MDD, aged 14–27 years. The study includes monthly assessment points over 18 months. The study includes a 48-month recruitment period and an 18-month treatment phase. The primary outcome is depressive relapse at 18 months, as measured by the Structured Clinical Interview for the Diagnostic and Statistical Manual of Mental Disorders, Fifth Edition (DSM-5), Research Version (SCID-5-RV). Secondary outcomes include the severity of depressive symptoms, time to relapse, time to remission, remission status, severity of anxiety symptoms, study and employment outcomes and cost-effectiveness. We will also examine four therapeutic mechanisms (mindfulness, self-compassion skills, social support and reduced rumination) to understand the ‘how and why’ of the intervention effects.

**Ethics and dissemination:**

Melbourne Health Human Research Ethics Committee (HREC/42967/MH-2018) provided ethics approval for this study. Findings will be made available through scientific journals and forums and to the public via social media and the Orygen website.

**Trial registration number:**

ANZCTR, ACTRN12619001412123.

**STRENGTHS AND LIMITATIONS OF THIS STUDY**

- *Rebound* is the first intervention to harness scalable digital technology to deliver a mindfulness-based intervention to prevent depressive relapse and manage depressive symptoms in youth diagnosed with major depressive disorder (MDD).
- *Rebound* was developed by a multidisciplinary team in partnership with young people and clinicians, which may enhance the acceptability of the intervention.
- The purpose of *Rebound* is to scale across and embed within youth mental health services.
- The *Rebound* study is the longest known study (18 months) of a digital intervention addressing relapse in youth diagnosed with MDD.
- Due to the nature of psychosocial interventions, participants and clinicians were not masked to treatment allocation.

##  Introduction

Adolescence and young adulthood are a time of forming social connections, exploring identity and engaging in work and study.^1^ Alongside already significant physical and emotional development, the onset and recurrence of major depressive disorder (MDD) can significantly affect a young person’s social functioning, physical health and quality of life.^2^ MDD is a common and often recurrent mental health condition, affecting approximately 12% of young people aged between 15 and 24 years.^3 4^ Globally, depression is the leading cause of disability^5^ and has increased in prevalence since the COVID-19 pandemic.^6^ Around 80% of children and young people accessing mental health services have a diagnosis of MDD.^7^ Depressive symptoms, alongside anxiety, represent the most common presenting problem of those accessing Australian, entry-level youth mental health services like headspace.^8^ While most young people recover from their first episode of MDD, around two-thirds will experience a relapse.^9^ The course of MDD shows a worsening pattern, with each episode occurring sooner, increasing in severity and becoming more resistant to effective treatments.^10^ Therefore, treatments must be timely, efficient and effective for those experiencing their first episode or who are at risk of subsequent episodes of depression. Unfortunately, even in high-income countries like Australia, the mental health system offers the opposite: delayed access to care due to limited availability of services and long waitlists and while in care, insufficient or mismatched treatments as indicated by many young people not reaching clinically significant improvement by the end of care.^8 11^

Digital mental health interventions may help to address systemic challenges by providing timely, accessible, evidence-based treatments.^12^ Digital interventions can be offered to young people while waiting for face-to-face care, during care, and on discharge and can extend the in-person ‘therapy hour’ by building on skills and concepts introduced in therapy.^13^ This means that digital interventions can treat current and emerging mental health disorders and support relapse prevention after remission. Moreover, digital mental health interventions are feasible and acceptable for use with young people experiencing depression.^14^ Digital health interventions have been shown to reduce the rate of hospital admissions and emergency service use and be both cost-saving and cost-effective for young people with serious mental ill-health over 18 months compared with treatment as usual (TAU).^15 16^

Despite the benefits of digital mental health interventions, engagement with and completion of online therapy content tend to be low.^17^ Real-time or ‘synchronous’ human support from a trained mental health professional improves engagement rates as mental health professionals can provide supportive accountability and motivational support.^18 19^ Furthermore, to provide an effective intervention, human support needs to lead to ‘effective’ engagement, which includes behaviour change.^20^ Behaviour change may also be supported by trained mental health professionals through the application of behaviour change techniques. Self-determination theory (SDT) is a theory of motivation that posits individuals are motivated in the most sustainable and healthy ways when they experience optimal levels of autonomy, competence and relatedness.^21^ SDT has been operationalised into evidence-based behaviour change techniques that can be applied to the provision of human support online.^22^

*Rebound* is a complex digital mental health intervention^23^ as it targets youth depression and prevents depressive relapse through the integration of several interacting components: (1) youth-friendly, evidence-based therapeutic content targeting mechanisms of mindfulness, self-compassion and rumination; (2) three types of human support (clinicians, peer workers and career consultants) applying evidence-based SDT-informed behaviour change techniques; and (3) a supportive online community of peers and peer workers sharing lived and living experience of mental ill-health, who aim to increase social support and decrease loneliness. *Rebound* has been iteratively developed with young people and a multidisciplinary team of clinicians, creative writers and developers over the past decade, using the Moderated Online Social Therapy (MOST) model.^24^,^26^ In a single-arm pilot trial (n=42), *Rebound* has been found to be safe, acceptable, and feasible and significantly improved depressive symptoms at a 12-week follow-up.^14^

The primary aims of the current study are:

1. To evaluate, via a randomised controlled trial (RCT), the effectiveness of the *Rebound* platform in preventing relapse of MDD in young people with MDD (aged 14–27 years).
2. To evaluate the cost-effectiveness of the *Rebound* platform via a concurrent within-trial economic evaluation.
3. To examine four therapeutic mechanisms (mindfulness skills, self-compassion, social support and reduced rumination).

The primary hypothesis is that relative to enhanced TAU, TAU plus *Rebound* will reduce the accumulated relapse rate over 18 months among young people with MDD. The secondary hypotheses are that relative to enhanced TAU, TAU plus *Rebound* will generate improvements in depressive symptoms, reduce time to relapse and time to remission, reduce remission rates, improve anxiety symptoms, and improve study and employment outcomes over 18 months; be more cost-effective; and help to prevent relapse in MDD by increasing mindfulness skills, self-compassion, social support and reducing rumination.

## Method

### Study design

The study design is a prospective, parallel group, rater-masked RCT. Approximately 255 participants with MDD will be allocated to either enhanced TAU or TAU in tandem with a complex moderated online social media intervention (TAU+*Rebound*).

The trial includes a 48-month recruitment period and an 18-month treatment phase, with the study being completed within 5.5 years. The design comprises monthly assessment points across 18 months. The protocol development addressed all aspects of Good Clinical Practice,^27^ CONSORT EHEALTH criteria^28^ and SPIRIT guidelines.^29^

### Setting

Participant recruitment commenced in October 2019 at services in the North-Western Melbourne (Victoria, Australia) catchment, specifically the Youth Mood Clinic (YMC), a programme of Orygen Specialist Program (OSP) and headspace centres led by Orygen (in the Melbourne suburbs of Sunshine and Glenroy).^30 31^ Orygen is the world’s leading research and knowledge translation organisation focusing on mental ill-health in young people.

Orygen Digital, the digital mental health division of Orygen and Centre for Youth Mental Health at University of Melbourne, designs, delivers and evaluates evidenced-based digital services for youth mental health. In April 2020, Orygen Digital commenced implementation of the MOST platform across all Victorian youth mental health services as part of the Victorian State Government’s response to the COVID-19 pandemic and the recommendations of the Royal Commission into Victorian Mental Health system.^11^ Due to the overlap between study and implementation sites, participant recruitment from Victorian sites ceased in October 2020. Instead, satellite recruitment sites were established at headspace services in the Illawarra region, New South Wales (NSW), operated by Grand Pacific Health (Wollongong, Bega, Nowra, Goulburn) and two services within the South Eastern Sydney Local Health District (Bondi Junction Community Mental Health Centre and headspace Bondi Junction).

### Patient and public involvement

The *Rebound* intervention has been co-designed with young people, following strict participatory design principles^32^ with continual feedback from young people across the development, pilot and intervention period. Consistent with best practice in developing novel interventions,^33^ we have obtained feedback from participants in the *Rebound* pilot^14^ about the need for more visually engaging therapy content. As a result, we have incorporated graphic narratives and comics to enhance engagement with therapy content in *Rebound*. Participants in the intervention group will be invited to regular focus groups with the emphasis being on feedback and questions about the intervention. More broadly, the protocol and participant information and consent forms have been reviewed by the Orygen Youth Research Council, and the investigator group carefully considered the burden of the trial schedule of assessments on participants.

### Participants

Inclusion criteria for participants are: (a) age 14 to 27 years inclusive; (b) able to read and converse in English; (c) able to provide informed consent; (d) able and willing to nominate an emergency contact person, such as a close family member; (e) diagnosis of MDD (current, partial or full remission) corresponding to the current episode of care as measured by the SCID-5-RV;^34^ and (f) ≥ 1 episode of MDD if in partial remission at time of screening assessment or ≥2 episodes of MDD (including the current episode) if current MDD or MDD in full remission at time of screening assessment.

Exclusion criteria are: (a) inability to converse in or read English; (b) acute risk of self-harm requiring urgent intervention (ie, suicidal ideation with a current plan and intent to enact this plan) at time of screening assessment; (c) a diagnosed permanent developmental delay or intellectual disability; (d) current or past episode of mania or hypomania; and (e) previous exposure to a MOST platform.

### Enrolment and randomisation

Participants are recruited from primary and specialist youth mental health services. In Victoria, study research assistants (RAs) attend weekly clinical review meetings at YMC and headspace services to identify eligible clients. In NSW, a study clinical liaison has been appointed at satellite sites to actively facilitate recruitment at the site. The study liaison attends clinical review meetings, engages with treating clinicians and screens clinical files to identify potentially eligible clients. The study liaison introduces the trial to the client and obtains consent to share contact details with Orygen. Young people can also self-refer via the study recruitment page on the Orygen website.

The RA contacts potential participants, provides a detailed explanation about the trial and offers to answer questions. All participants are required to provide informed, signed consent. Parental or legal guardian consent is required for participants under 18 years of age. Once consent is obtained, the participant is enrolled in the trial and a screening assessment to assess eligibility is conducted by the RA. Once eligibility is established, the baseline assessment is completed. After completing the baseline assessment, the participant is randomised by the RA via a secure online Research Project Management System (RPMS). The RPMS sends an automated email to the study coordinator and principal investigator, notifying them of the outcome of randomisation. The study coordinator informs the participant of the allocation. Participants are reimbursed for their time for each assessment completed.

The randomisation schedule is generated by a statistician independent of the study, programmed in the RPMS and not accessible by the study team. Participants are randomly assigned to the treatment condition via the RPMS using randomly permutated blocks with a 1:1 allocation ratio. Participants are stratified by current MDD status (current, partial or full remission), number of previous MDD episodes (≤ 2 or ≥ 3), age (<18 years and ≥18 years), sex at birth and by treatment centre.

### Enhanced treatment as usual (TAU)

TAU consists of a range of treatment options delivered by the treating service prior to discharge^30 31^ and/or generic medical or mental health services typically available to young people in the absence of enrolment in the study. These can include follow-up by a general practitioner, private psychiatrist, primary care youth mental health services or adult mental health services, which deliver multidisciplinary psychiatric care (including medical follow-up, case management and acute psychiatric care as appropriate). Online psychoeducation is also offered to those allocated to enhanced TAU to match the additional support offered to the intervention group. Psychoeducation is beneficial as an adjunctive treatment for depression and is readily translated into web-format.^35^ The study team developed a web application called ‘Empower Your Mood’ (EYM) that includes psychoeducation modules on depression symptoms, causes and course, behaviour and mood, information on diet, exercise, sleep, social support and getting support. In contrast to the *Rebound* platform, EYM is a static website and does not include clinician support or an online community. All modules are available for 18 months to match the *Rebound* intervention’s timeframe.

### Intervention

*Rebound* is a complex, multicomponent intervention^23^ based on the MOST model.^1415 24^,^26^ The design of *Rebound* considers SDT principles to best support intrinsic motivation^36^ (see table 1). Each component of *Rebound* will be introduced in turn.

**Table 1:** Self-determination theory (SDT)-informed platform features

SDT principle	Brief definition	Moderated Online Social Therapy platform feature
Autonomy	Freely chosen actions, including genuine interest, value and appreciation for the activity	Personalised approach and flexibility in support and content options
Competence	A sense of growth through progressive mastery over a task	Bite-sized therapeutic content, demonstrating progress in content completion, toolkit of saved items
Relatedness	Meaningful and supportive bi-directional social connections	Online community and talking points

#### Therapeutic content

**Figure 1:**
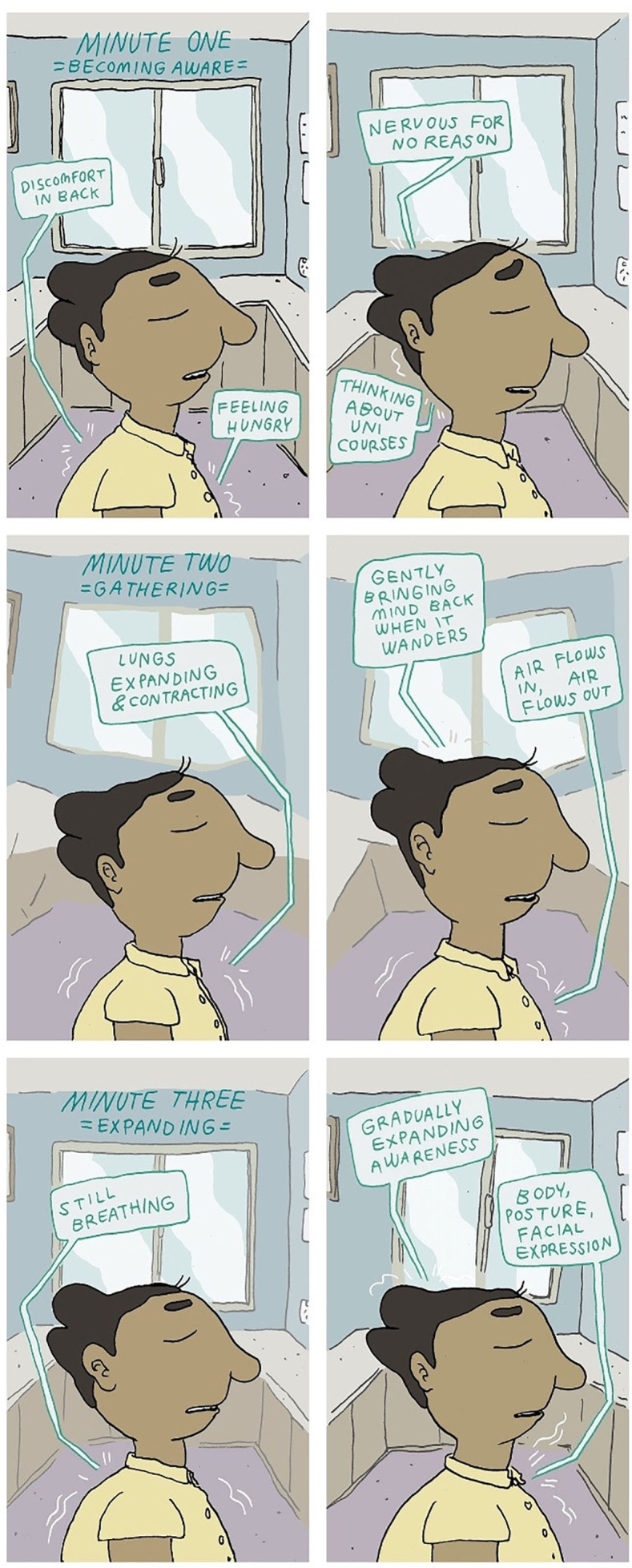
Excerpt of the ‘Three Minute Breathing Space’ comic.

To scaffold the building of competence, the therapeutic content on *Rebound* is presented in ‘bite-size’ chunks—thematically related ‘activities’ that are organised into ‘tracks’—that are clustered within ‘journeys’. There are five activity types: *comics*, *reflective actions*, *actions*, *talking points* and *pages. Comics* are illustrated multipaneled narratives that bring therapeutic concepts to life via recurring characters and story (see figure 1 for an excerpt), *reflective actions* provide a clear prompt for reflection, *actions* suggest a practical step (eg, behavioural experiment), *talking points* prompt young people and peer workers to post their thoughts and reactions to the content, and *pages* summarise each track and provide psychoeducation. Users have the option to save activities to a ‘toolkit’, so they have an accessible, personalised and labelled bank of strategies when needed.

*Rebound* includes five ‘journeys’, each with a different focus: managing depression (‘Improve Your Mood’), managing anxiety (‘Find Your Calm’), overcoming social anxiety (‘Find Your Confidence’), managing insomnia (‘Feeling Tired’) and enhancing social functioning (‘Social Hacks’). There is also one journey based on work and study-related issues. Users can access one journey at a time and on-demand ‘Explore’ activities alongside their journey.

Participants randomised to the TAU+*Rebound* condition are initially offered the Improve Your Mood journey, which adopts a trans-therapeutical approach to depressive relapse prevention using Mindfulness-Based Cognitive Therapy, Acceptance and Commitment Therapy and Cognitive Behavioural Therapy principles. The Improve Your Mood journey contains over 50 activities, which are available over the 18 months of the trial. The Improve Your Mood journey targets rumination and worry, behavioural inactivation and avoidance by introducing the following strategies: mindfulness, behavioural activation, values, defusion, cognitive restructuring, acceptance, self-compassion and gratitude.

#### Peer-to-peer online social networking (‘the community’)

**Figure 2:**
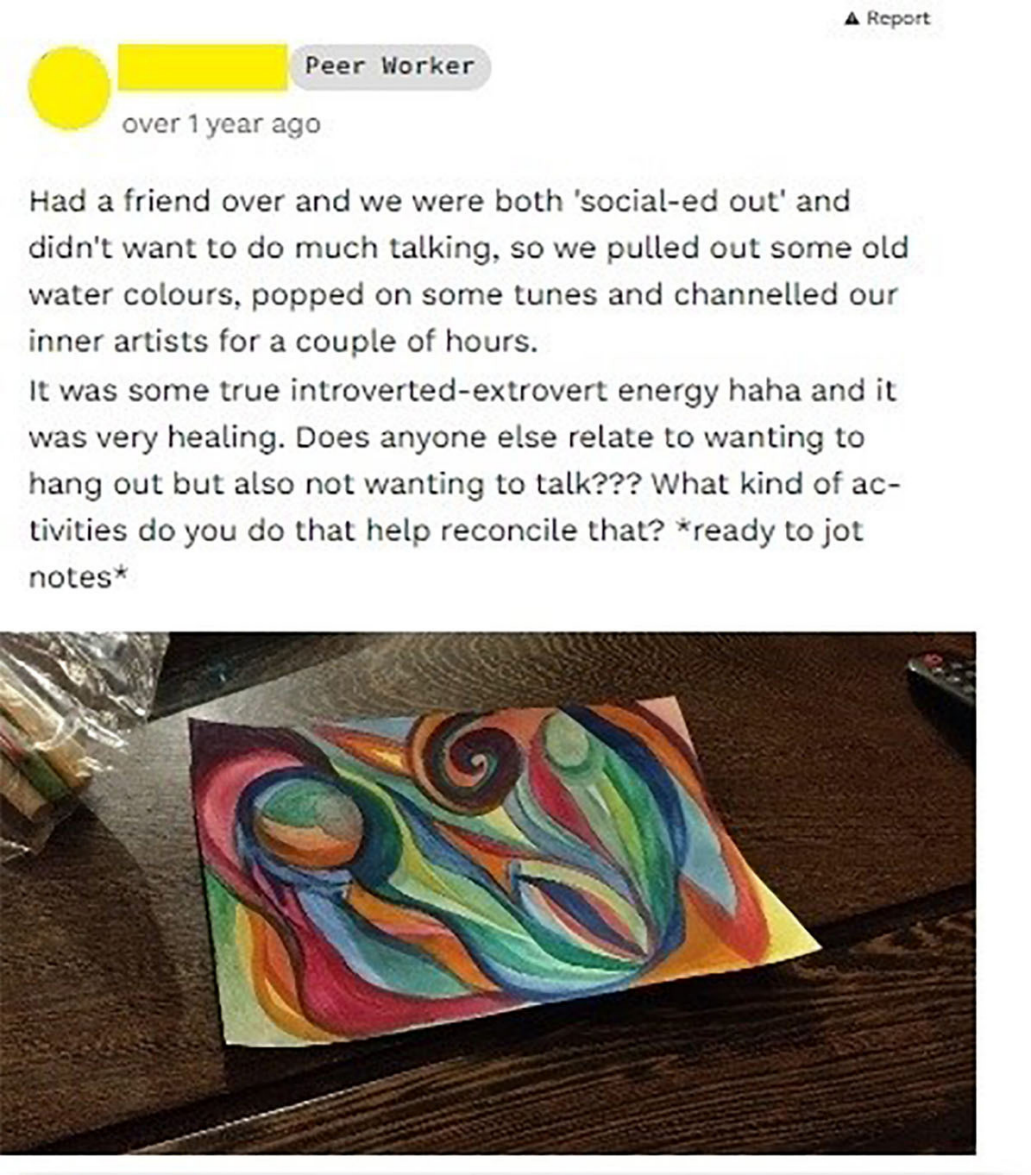
An example community post (de-identified and used with permission).

The online ‘community’ allows young people and peer workers to develop a profile listing their interests (‘members’ page) and to post text, images and links (‘feed’ page). On the community feed, peer workers post regularly about their lived and living experience of mental health (see figure 2) and contribute ice breaker–type posts about their interests. When contributing a post to the feed, users also have the option to apply the ‘vent’ function to the post, which allows users to include certain offensive words (most commonly swear words) that would otherwise be blocked by the MOST system. Vent posts are by default collapsed in the feed so that their viewing is optional, and other users who wish to see such posts are required to click expand to view them. Users may ‘react’ (eg, ‘I get you’, ‘thinking of you’) or comment in response to a post. The community is designed to facilitate social support and enhance relatedness.

The community also includes Talk it Out (TiO), a structured problem-solving function that steps a young person through developing a question (eg, How can I manage stress?), provides an opportunity for the young person to crowdsource solutions and checks in with their progress. TiO was informed by the Social Problem-Solving Framework.^37^

#### Human support

There are three types of human support (or ‘moderation’) offered via *Rebound*: clinicians, peer workers and career consultants. These moderators work together to provide care for each user of *Rebound* using SDT-informed engagement strategies (see table 2). *Rebound* moderators meet fortnightly as a whole team and weekly in discipline-specific groups to review engagement on the platform and revise SDT-related strategies to support motivation. Moderators can also communicate with one another via chat messaging and activity logs on *Rebound*.

**Table 2:** Self-determination theory (SDT)-informed engagement strategies^22^

SDT concept	Strategies^22^	Relevance to moderation on *Rebound*
Autonomy	Use non-judgemental language that suggests, rather than demands.Note sources of internal and external pressure.Encourage the user to develop their own understanding of their mental health.Provide a clear rationale for suggestions.Explore the user’s values and aspirations.Provide choices (including the choice not to change).Encourage experimentation with new skills, with debriefing.	Clinical formulation development.Encouraging reflection in 1:1 interactions.Moderation team being mindful to not place pressure on user in interactions.Explore sources of internal/external pressure in 1:1 interactions, particularly with peer work/clinicians.Create culture within moderation team of speaking to one another non-judgementally and then spread culture to platform.Apply strategies to goal setting on platform.Link journey/platform engagement to values.Develop shared rationale for journey/platform engagement.Provide choices about goal setting, journey tailoring, mode and frequency of contact.Support engagement in reflective actions.Encourage light, fun and experimental actions.
Competence	Discuss barriers and how to overcome them.Clarify goals.Support the user to set realistic goals.Validate progress being made.Support the user to develop a plan to meet their goals.Encourage the user to self-monitor progress.Explore dealing with unhelpful internal and external pressure.	Ensure user knows that you are figuring out the barriers together—reduces power imbalance.Complete part of journey with user to identify and troubleshoot barriers in real time.Clinicians help user to identify what they expect of themselves and encourage consideration of what they have capacity for.Peer workers share experiences of process of change.Clinicians and career consultants help user to set personalised and realistic goals on a realistic timeline.Clarify differences between long-term and short-term goals.Clarify differences between goal-setting on MOST vs other types of support (eg, face-to-face).Career consultants provide feedback on resumes and cover letters.Clinicians provide encouragement and validation when progress made (eg, completion of activities).Clinicians provide feedback on reflective actions and skill development (eg, mindfulness); user identifies strengths through questionnaire, and moderators can identify and point out strengths in action.Setting realistic amounts of activity on platform per day (eg, 2 min, one activity) in order to meet longer term goals (eg, complete journey).Using non-controlling language, acknowledging the present ‘capacity’ of the user.Reminding them of when they have overcome pressure in the past.Encouraging them to explore what is internal/external pressure.Ask questions to prompt reflection on why they are doing what they're doing—align with core values.Provide a rationale for why we are asking user to engage.Acknowledge pressure that can be experienced.Provide information about work rights.Encourage boundaries.
Relatedness	Respect the user’s perspective.Encourage the user to ask questions.Demonstrate unconditional positive regard.Demonstrate genuine interest in the user.Listen empathetically.Check the user’s available supports and link them in to support where needed.	Provide validation and empathy in all interactions.Validate before jumping to problem-solving.Encourage user to ask questions of moderators and in the community, particularly using TalkitOut.Encourage self-reflection on progress.Build trust so that user feel comfortable asking questions.Regularly ask if user have any questions.Unconditional positive regard in all interactions.Genuine interest in all interactions.Offer choices for types of support.Check in on supports later in user’s time on platform.Assess supports during welcome call/initial interactions with user.Career consultants refer user to external supports and resources as needed.

*Clinicians*. Each young person on *Rebound* is allocated a qualified mental health clinician (eg, clinical psychologist, social worker) with experience working with young people. During the initial ‘welcome call’, clinicians welcome young people to the platform, provide a platform orientation, support goal setting and develop a formulation. After this, clinicians contact the young person weekly for 12 months (via phone/SMS/chat) to support engagement with therapy content, suggest and tailor therapy content, and act as a liaison between the young person and other forms of human support. In their final 6 months, clinicians reduce contact to monthly.

To maintain usability, relevance and uptake of content, clinical moderators tailor journey content to each user’s immediate needs. Tailoring is also based on the user’s remission status. For example, for participants who are actively depressed, activities related to behavioural activation/motivation, sleep hygiene and avoidance may be prioritised, while those in full remission may focus on mindfulness, values/strengths, wellness planning and developing their toolkit. Cognitive strategies (ie, restructuring, defusion) and self-compassion are addressed regardless of remission status. Users may also choose to switch to a different journey in consultation with their allocated clinician.

Importantly, the clinician monitors the clinical status of young people in their caseload and conducts twice-daily safety checks of the platform. The clinical team meet weekly for peer supervision. Clinicians complete fidelity checklists bi-monthly and discuss their self-identified strengths and weaknesses during supervision to strengthen fidelity to the moderation model and processes.

*Peer workers*. Peer workers are trained young people with lived and living experience of mental ill-health. Peer workers are integral to the provision of social support via *Rebound*—a proposed mechanism of action in the study (eg,^38^ Peer workers moderate the online ‘community’ and chat with young people on the platform. Peer workers also lead monthly online ‘hangouts’ to reinforce connections. The peer work model is also designed to normalise experiences, counteract stigma, and promote platform engagement.

*Career consultants*. Career consultants provide specialised support for work and study issues via phone and chat including support with job applications, interviews and career assessments.

### Safety protocol

The safety protocol is comprised of three levels of security including: (1) system and privacy protection; (2) online safety and (3) clinical safety.

The *Rebound* platform is hosted on an Amazon Web Services web server. Amazon Web Services and Orygen Digital meet standards of responsible business practice. Identity management and networking are handled by Orygen Digital’s Engineering Department according to Orygen (National) Standards, which meet Australian research requirements. In addition, MOST has a wide range of measures to secure the application and database against unauthorised access. These measures conform to industry best practice as defined by the Open Web Application Security Project (www.OWASP.org). Privacy and online safety are managed in accordance with the Australian Communications and Media Authority.

At onboarding, the *Rebound* clinician carries out an initial orientation with *Rebound* participants, including details of the terms of use. Participants are required to accept and comply with the guidelines for safe use of *Rebound*. When needed, participants are offered guidance on appropriate usage of the system. All users are asked to nominate an emergency contact person, such as a close family member. *Rebound* includes a ‘report function’ that enables young people to report a concern about any material posted by a user. The moderator assesses the report and responds accordingly, which may include removing material and, in some cases, deactivating or restricting the poster person’s account. Participants are also able to hide their profile and activity should they become concerned about their privacy.

Clinical risk is managed through both manual and automated procedures. First, clinical moderators monitor the system two times per day on weekdays and one time per day on weekends for evidence of clinical risk or deterioration. Any detected increased risk activates the *Rebound* risk and safety protocol, which includes one or more of the following: a risk assessment with the young person, informing the research team, alerting the emergency contact nominated by the participant and liaising with suitable emergency services where necessary. In addition, the system incorporates visible emergency guidelines and contact information. Finally, *Rebound* includes an automated keyword detection function, which activates each time a participant posts a contribution indicative of clinical risk or that contains potentially offensive words. The function blocks posts with notifications sent to the young person and the moderator, who can ‘unblock’ the post should they determine it unproblematic.

In the event of a clinically significant deterioration of symptoms, increased risk of suicide or a hospital admission, a clinical moderator performs an assessment to determine the risks and benefits of a temporary withdrawal from *Rebound*. Based on this assessment, and in consultation with the young person, the clinical moderation team determines whether their account is temporarily suspended, or their level of access is restricted. Following suspensions or restrictions to a user’s account, the clinician will contact the young person at monthly intervals to ascertain whether the account is to be reactivated.

### Outcome measures

Multiple methods will be employed to assess study outcomes (see table 3). These include independent observer ratings, self-report and usage data from the *Rebound* system.

**Table 3:** Schedule of outcomes and measures

	Screening/baseline	3 months	6 months	9 months	12 months	15 months	18 months	
**Primary outcome and measure**								
Relapse of MDD (SCID-5-RV: depression module)	x	x	x	x	x	x	x	
**Secondary outcomes and measures**								
Depressive symptoms (QIDS-SR)	Monthly							
Time to relapse (SCID-5-RV: depression module)	x	x	x	x	x	x	x	
Time to remission (SCID-5-RV: depression module)	x	x	x	x	x	x	x	
Rate of remission (SCID-5-RV: depression module)	x	x	x	x	x	x	x	
Time in remission (SCID-5-RV: depression module)	x	x	x	x	x	x	x	
Anxiety (GAD-7)	x		x		x		x	
Vocational status (RUQ)	x		x		x		x	
Cost-effectiveness (RUQ)	x		x		x		x	
**Therapeutic mechanisms and measures**								
Mindfulness skills (FMI)	x		x		x		x	
Self-compassion (SCS-SF)	x		x		x		x	
Social support (SPS)	x		x		x		x	
Rumination (RRS-SF)	x		x		x		x	
**Exploratory outcomes and measures**								
Stress (PSS)	x		x		x		x	
Social anxiety (LSAS)	x		x		x		x	
Loneliness (UCLA Loneliness Scale)	x		x		x		x	
Self-efficacy (Self-Efficacy Scale)	x		x		x		x	
Self-esteem (Rosenberg SES)	x		x		x		x	
Social and general functioning (SOFAS)	x		x		x		x	
Suicidal ideation and attempts in past 6 months (CSSRS)	x		x		x		x	
Deliberate self-harm (Risk Taking and Self-Harm Inventory†)	x		x		x		x	
Psychological well-being (BPNSS)	x		x		x		x	
Quality of life (AQoL-8D)	x		x		x		x	
Behavioural activation (BADS-SF)	x		x		x		x	
Sleep quality (PSQI)	x		x		x		x	
Strengths use (SUS)	x		x		x		x	
Medication adherence (RUQ)*	x		x		x		x	
Substance use (ASSIST)*	x		x		x		x	
Digital phenotype (AWARE-Light)	Continuous							
Affective and cognitive reactivity and social support (SEMA3 application)	Daily							
**Intervention group-only outcomes and measures**								
Intervention acceptability (qualitative evaluation via semistructured interview)							x	
Therapeutic alliance (WAI-C, WAI-T)	x		x		x		x	
Intervention use (ie, frequency, duration, pattern)	Continuous							

* Potential covariates.

† Risk Taking and Self-Harm Inventory for Adolescents.

AQoL-8DAssessment of Quality of Life - 8 DimensionsASSISTAlcohol, Smoking and Substance Involvement Screening Test BADS-SFBehavioural Activation for Depression Scale-short formBPNSSBasic Psychological Needs Satisfaction ScaleCSSRSColumbia-Suicide Severity Rating ScaleFMIFreiburg Mindfulness InventoryGAD-7Generalized Anxiety Disorder 7-item scaleLSASLiebowitz Social Anxiety ScaleMDDmajor depressive disorderPSQIPittsburgh Sleep Quality IndexPSSPerceived Stress ScaleQIDS-SRQuick Inventory of Depressive Symptomatology-Self-ReportRRS-SFRuminative Responses Scale - short formRUQResource Use QuestionnaireSCID-5-RVStructured Clinical Interview for DSM-5 Disorders, Research VersionSCS-SFSelf-Compassion Scale Short FormSEMA3Smartphone Ecological Momentary AssessmentSESSelf-Esteem ScaleSOFASSocial and Occupational Functioning Assessment ScaleSPSSocial Provisions ScaleSUSStrengths Use ScaleUCLAUCLA Loneliness Scale (Version 3)WAI-CWorking Alliance Inventory client versionWAI-TWorking Alliance Inventory therapist version

#### Primary outcome and measure

The primary outcome is *relapse rate* (accumulated over 18 months) of MDD. Relapse will be defined as a return, for at least 2 weeks, of symptoms sufficient to meet DSM-5 criteria for MDD (as determined by the SCID-5-RV)^34^ at any time in the 18 months post randomisation. This definition of relapse is the one most commonly used in clinical studies.^39^ Measurement of relapse will be carried out every 3 months (ie, at 3, 6, 9, 12, 15 and 18 months). Telephone administration of the SCID is a valid method for measuring MDD.^40^ Data from the Quick Inventory of Depressive Symptomatology Self-Report^41^ may also be used to supplement the SCID assessment. A number of strategies will be embedded to promote participant retention, including a generous assessment window, flexible availability of RAs, minimal data collection at most timepoints and honing RA interview skills to minimise time commitment at larger assessment points.

#### Secondary outcomes and measures

Secondary outcomes and measures include:

1. *Depressive* symptoms will be measured by the QIDS self-report instrument^41^ monthly for 18 months.
2. *Time to relapse* will be derived from the SCID-5-RV (Module A: Mood Episodes) at 3, 6, 9 12, 15 and 18 months.
3. *Time to remission* will be derived from the SCID-5-RV (Module A – Mood Episodes) at 3, 6, 9, 12, 15 and 18 months.
4. *Rate of remission* will be derived from the SCID-5-RV (Module A – Mood Episodes) at 3, 6, 9, 12, 15 and 18 months.
5. *Time in remission* will be derived from the SCID-5-RV (Module A: Mood Episodes) at 3, 6, 9, 12, 15 and 18 months.
6. *Anxiety* will be measured by the Generalized Anxiety Disorder 7-item scale^42^ at baseline, 6, 12 and 18 months.
7. *Vocational status* will be self-reported by participants at baseline, 6, 12 and 18 months (including the participant’s report of employment and educational activities in between assessments).
8. *Cost-effectiveness* will be assessed using a self-reported Resource Use Questionnaire (RUQ) to determine the broader resource use of participants (eg, community mental health services, hospitalisations, work and educational impacts) and the Assessment of Quality of Life - 8 Dimensions (AQoL-8D) questionnaire, which measures health-related quality of life and can be used to calculate quality-adjusted life years (QALYs).^43^

#### Measures of therapeutic mechanisms

Therapeutic mechanisms include: *mindfulness skills*, measured by the Freiburg Mindfulness Inventory;^44^ *self-compassion,* measured by the Self-Compassion Scale Short Form;^45^ *rumination*, measured by the Ruminative Responses Scale-Short Form;^46^ and *social support,* measured using the Social Provisions Scale.^47^ Measures are administered at baseline, 6, 12 and 18 months.

#### Other measured outcomes

Additional exploratory outcomes, potential covariates and intervention-only outcomes are outlined in table 3.

### Data analysis and management

#### Sample size

Sample size was determined by power analysis using G*Power 3. The primary outcome is difference in accumulated relapse rate over 18 months. A previous relapse prevention study in youth MDD reported a relapse rate at an 18-month follow-up of 62% for TAU and 36% in the relapse prevention group (OR=2.9).^48^ Since *Rebound* will be compared against an enhanced TAU, we assume a smaller effect (OR=2.5), for which a total sample of 192 people is required to achieve 85% power (alpha=0.05). A total of 255 participants will be recruited, allowing for a 25% attrition rate. This compares favourably with the attrition rates in the *Rebound* pilot (7%) and is comparable at an 18-month follow-up to the Horyzons RCT (25%).^14 15^

#### Data management

The RPMS is used to manage all outcome data. The RPMS includes an electronic Case Report Form (eCRF). The RAs record participant-level data on an eCRF. These data are subsequently entered into the eCRF section of the RPMS. Self-report data are entered directly by the participant. The RPMS is accessed using a secure website and is stored on a secure server. It is designed to maintain the privacy and confidentiality of participant information and to ensure the integrity of the data. Access to RPMS is restricted to study personnel, and the level of access is dependent on the person’s role.

#### Analysis of outcomes

Primary analyses will be undertaken on an intention-to-treat basis. Group differences in accumulated relapse rate at 18 months (primary outcome) will be tested using Fisher’s exact test. The Cox proportional hazards regression model (survival analysis) will be used to model time to relapse and rate of remission in each group, which will also be used to derive the hazard function reflecting the instantaneous probability of relapse/remission at any time over the 18-month follow-up.^49^ Finally, group differences (*Rebound* plus TAU vs enhanced TAU) in change over time in the continuous secondary outcomes across the 18-month follow-up will be examined using random-effects models. Random-effects models allow for estimation of between-person differences (ie, group effects) in within-person slopes (ie, change trajectories in secondary outcomes) and are the preferred methods for analysing clinical trial data.^50^

Mechanism of action analyses will be conducted using a multilevel structural equation modelling framework to assess mediation.^51^ Specifically, person-specific slopes representing change over time in the proposed mechanism-of-action variables will be examined as mediators of the effect of treatment group (*Rebound* vs control) on risk of relapse.

Additional analyses will use multiple imputation to assess the robustness of the findings to the choice of method for handling missing data. Additional comparisons between treatment groups based on completers-only analyses will be conducted. Analyses will be undertaken in accordance with The International Council for Harmnonisation (ICH) 9 guidelines including a full analysis as well as per protocol set. The per protocol sample will be defined based on receiving a prespecified minimal exposure to the online intervention (ie, more than 16 logins over the 18-month intervention period).

The economic evaluation will comprise a cost-consequences analysis comparing the incremental costs of the *Rebound* platform (vs treatment as usual) to a wide range of incremental study outcomes (eg, QALYs, relapse rate). Inclusion of the AQoL-8D questionnaire facilitates the derivation of QALYs and enables a cost-utility analysis to be undertaken. A study-specific RUQ was adapted for this study from another RUQ frequently used in Australian mental health-related economic evaluations.^52^ The RUQ encompasses: community-based health service use; hospitalisations; accommodation services; medication and diagnostic tests; impacts on education and employment; and other relevant services. Best practice within-trial economic evaluation methods will be adopted^53^; and the comparative cost-effectiveness of the *Rebound* platform (vs treatment as usual) will be summarised using the incremental cost-effectiveness ratio metric (eg, cost per QALY). If the *Rebound* platform is found to be effective, then the lifetime and population cost-effectiveness of the intervention will be evaluated using modelling techniques.

#### Monitoring

Adverse events will be recorded throughout the trial and serious adverse events will be reported to the sponsor (Orygen). The study was assessed as low risk by the sponsor, and a trial management group will be established in place of a data monitoring committee. The sponsor monitoring plan includes an initial monitoring visit early in the recruitment phase which will determine the ongoing monitoring schedule. Discontinuation of the study will be considered where:

- A participant attempts suicide, and it is highly likely that the attempt was caused by the Rebound intervention.
- There are repeated instances of participants notifying moderators about triggering or distressing content posted by other users.

Decisions about study discontinuation will be determined by the trial management group in consultation with the sponsor.

## Discussion

Accessible, timely and evidence-based treatments are imperative to meet the demands of the present youth mental health crisis.^4 54^ Rebound is a complex digital intervention;^23^ unique in its length (18 months); engagement principles (using SDT-informed strategies and a multidisciplinary approach); and presentation of youth-friendly, third-wave mindfulness-based content. It is the first accessible, scalable treatment to prevent relapse in youth MDD, addressing a major gap in public youth mental health services. The study includes: an active control group, an extended follow-up, cost-effectiveness analysis and evaluation of theory-driven mechanisms of action. This is likely to advance models of relapse in youth depression and inform future intervention development.

Furthermore, the findings of this study serve to advance our knowledge of digital adaptations of mindfulness-based approaches and how to best engage young people online and prevent depressive relapse. If the trial is successful, there is a clear translation pathway for findings to the rollout of MOST.^13^ Recruitment of trial participants was finalised in September 2023. Follow-up assessments will conclude in March 2025.

### Dissemination

Findings will be made available through scientific journals and forums and to the public via social media and the Orygen website. De-identified individual participant data will be available after publication for 3 years via the Health Data Australia catalogue (https://www.researchdata.edu.au/health). Requests must include a methodologically sound proposal. Specific conditions of use may apply and will be specified in a data sharing agreement (or similar) that the requester must agree to before access is granted (online supplemental file 1). Study protocol, informed consent material and statistical analysis plan will also be available.

## supplementary material

10.1136/bmjopen-2024-088695 online supplemental file 1
