# Supplementary material for: A mindfulness-based, cognitive, social, digital relapse-prevention intervention for youth with depression in Australia: study protocol for a randomised controlled trial of Rebound
Source: BMJ Open. 2024 Nov 27;14(11):e088695. doi: 10.1136/bmjopen-2024-088695 (PMC11603739; doi:10.1136/bmjopen-2024-088695)
Supplement: online supplemental file 1 [file bmjopen-14-11-s001.docx]

*
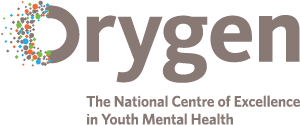

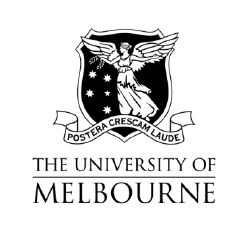
*

**Participant Information Sheet/Consent Form**

**Interventional Study** - *Adult providing own consent*

| **Title** | Preventing relapse of major depressive disorder in youth: RCT of a novel mindfulness-based cognitive online social therapy |
| --- | --- |
| **Short Title** | Rebound |
| **Project Number** | 2018.217 |
| **Project Sponsor** | Orygen National |
| **Coordinating Principal Investigator** | Professor Mario Alvarez |
| **Principal Investigator** | (Insert Site PI) |
| **Study Coordinator** | Mrs Daniela Cagliarini |
| **Location** | - (Insert locations) |

**Part 1 What does my participation involve?**

**1 Introduction**

You are invited to take part in this research project. This is because you are receiving treatment at (site) and have been identified as having experienced depression.

The aim of this research project is to test a new website designed to assist young people in their recovery from depression. The new website is called Rebound.

This Participant Information Sheet/Consent Form tells you about the research project. It explains the assessments and treatments involved. Knowing what is involved will help you decide if you want to take part in the research.

Please read this information carefully. Ask questions about anything that you don’t understand or want to know more about. Before deciding whether or not to take part, you might want to talk about it with a relative, friend, your case manager or your doctor.

**Your participation is voluntary**

Your participation in this study is completely voluntary and there will be no cost to you. If you do not want to take part in this study you do not have to. You should feel under no obligation to participate in this study. Choosing not to take part in this study will not affect your current and future medical care in any way.

If you decide you want to take part in the research project, you will be asked to sign the consent section. By signing it you are telling us that you:

• Understand what you have read

• Consent to take part in the research project

• Consent to have the tests and treatments that are described

• Consent to the use of your personal and health information as described.

You will be given a copy of this Participant Information and Consent Form to keep.

**2 What is the purpose of this research?**

The researchers have developed a new online program, called Rebound, for young people recovering from depression. The program was co-designed with young people who have experience of mental illness and has been successfully pilot tested. This means a small number of people tested Rebound so that we could find out if it was practical and safe to do a larger study. This program includes some of the features of websites that you may already be familiar with along with some important new features. For example, it allows the user to display their own profile and communicate online with other young people who have experienced depression, in the Rebound online ‘community’. This is a private online space which is only open to young people who have been treated at a participating centre.

There are online moderators in Rebound, based at Orygen National (Melbourne), whose job is to help users make the most of the system and to encourage a positive and supportive experience. In addition, there are peer support workers who are young people who have personal experience of mental illness and who are available in Rebound to provide encouragement and share their own experiences. Also it will be up to each user whether or not they use their real name but everyone on the system will be a real person, including the moderators.

As well as being a social networking website the program also includes lots of online therapy material. This therapy content has been specially designed to help young people recover from depression, and the aim is that users can complete these in their own time. The therapy journeys include information and interactive activities about depression, how to identify and use personal strengths, how to build your social confidence, how to stay well, and many other topics.

We have designed Rebound because we believe it is important to have a resource that young people recovering from depression can use when and where it is convenient for them. Also, we thought it would be helpful for young people if they could share their experiences and receive positive encouragement from others going through similar experiences.

Because we do not yet know if Rebound is effective in helping young people recover from depression, we are comparing Rebound with the effects of receiving online information about depression and recovery that doesn’t include therapist or peer support. In order to make this comparison we will be allocating 50% of participants in the study to Rebound plus usual care and 50% of participants in the study to receive the online information plus usual care. Each participant who consents to participate will have an equal chance of being in each of these two groups.

This research has been initiated by researchers from Orygen National, The Australian Catholic University, and The Department of Computing and Information Systems at The University of Melbourne. This research has been funded by a grant from The National Health and Medical Research Council (NHMRC).

**3 What does participation in this research involve?**

This trial is being conducted under the “Teletrials” model. This means that while you will receive your usual treatment at (site), your participation in the study will involve visits via teleconference or phone with study team members at Orygen National, the lead trial site.

Study Design

If you agree to participate you will be taking part in a randomised controlled research project. Sometimes we do not know which treatment is best for treating a condition. To find out we need to compare different treatments. We put people into groups and give each group a different treatment. The results are compared to see if one is better. To try to make sure the groups are the same, each participant is put into a group by chance (random). This is also a blind study, which means that the study research assistant will not know which group each participant has been allocated to. Your treating team will know which treatment you are receiving. If you have a local doctor, we strongly recommend that you inform them of your participation in this research project.

Consent

You will first have a meeting with the study research assistant, who will explain the study to you and answer any questions you may have. You will be provided with this document to take home and read. You will be given time to decide whether or not you would like to participate. You can talk to your treating team, your family or friends. If you do decide that you would like to take part, you will be asked to sign the consent form at the end of this document.

Screening and Baseline Assessment

To determine whether or not you are eligible for the study, you will be asked to take part in an interview with the study research assistant who will ask some questions about the symptoms you have been experiencing, as well as asking specific questions that will help them find out relevant information about you. These assessments and interviews are referred to as the screening assessment.

Once you have completed the screening assessment and this initial assessment indicates that you are eligible for this study, you will then complete what is referred to as a baseline assessment. The baseline assessment is a more thorough interview to help research study staff better understand the symptoms you have been experiencing and includes questions that

measure symptoms of depression, social wellbeing and functioning, psychological wellbeing, perceived stress levels, worry, quality of life, self-efficacy, social support, loneliness, satisfaction with life, sleep, use of personal strengths, and mindfulness skills. The research assistant will also ask about your use of medications, other substances, and health services. Together, the screening and baseline assessment is expected to take around 2 hours. You can take breaks as needed, or the interview can be scheduled over two appointments.

Group Allocation

Once the questionnaires and interviews have been completed you will be informed whether you have been allocated to Group 1 or Group 2. You will have an equal chance of being allocated to either group.

If you are one of the first participants to join the project you may not have access to Rebound immediately. Rebound will launch after enough people have been recruited in order to make sure that there is sufficient online activity.

Group 1

If you are allocated to Group 1 you will have access to all of your usual treatments in addition to having access to Rebound. Rebound will be available until the end of the study. Participants in this group will be provided with log-in details to access Rebound, and complete an online orientation to the system to help you get the most out of using Rebound. The moderator will then contact you via phone to answer any questions and discuss how you can make the most of Momentum.

You will be able to use Rebound from any computer, tablet or mobile phone that has internet access. It is up to you when and how often you log on and what you do when you log on. However, based on prior experience and research, we believe it is most helpful to spend a little bit of time on Rebound at least 4-5 times a week and to practice strategies at other times when you’re not online. If you stop using Rebound for a while we will call you to see how you are going. So that we can keep Rebound a safe and private program there will be some rules that all users will be expected to follow, such as being respectful to other users and keeping messages in Rebound confidential.

Please note that inappropriate use of the Rebound system (e.g. derogatory or disrespectful statements) may lead to your Rebound account being temporarily or permanently suspended. It is also important to note that online moderators will monitor Rebound twice a day during weekdays and once a day during weekends and on public holidays. Therefore, Rebound has not been designed and is not equipped to respond to emergency situations. Details of who to contact in an emergency are provided within the Rebound system.

Group 2

If you are allocated to Group 2 you will have access to all of your usual treatments in addition to having access to a website with information about depression and recovery, called Empower your Mood. This website will not include expert or peer moderation, or social networking features.

Follow up assessments

All participants, no matter which group they are in, will be asked to meet with the study research assistant to repeat some of the questionnaires at 6, 12 and 18 months after your initial study interview. These follow-up meetings will take approximately 1.5 hours. In addition, each participant will be asked to complete a short online assessment every month and a phone assessment every 3 months, in between face-to-face interviews, to assess symptoms of depression. These assessments will take up to 15 minutes.

Group 1 only assessments

If you are allocated to Group 1, you will be asked to complete a questionnaire about your online moderator one month after you get access to Rebound and again at 6, 12 and 18 months. You may also be asked to complete an interview about your experience of using Rebound at the end of the study. This interview will be audio recorded and transcribed into written text via a video conferencing platform.

SEMA

Participants owning a smartphone will also be asked to use a mobile application, called SEMA, which will administer a short questionnaire, including questions about current mood and social interactions, once per day for the first 12 months of your involvement in the study. The survey will be sent at the same time every evening at a time suitable to you. You will receive an initial notification to complete a questionnaire and up to 2 reminders approximately 45 and 88 minutes later. You will have up to 90 minutes to complete the survey. Each SEMA questionnaire will take approximately 2 minutes to complete. We understand that completing a survey each day may be not possible or desirable for all participants so you can let us know if you need to take a break from this.

Reimbursement

There are no additional costs associated with participating in this research project, nor will you be paid. All treatment and tests required as part of the research project will be provided to you free of charge.

At each of the four face-to-face interviews, you will be reimbursed $30 per hour for your time, up to a maximum of $60 per interview. Additionally, you will be reimbursed for time taken to complete the SEMA questionnaires (30c per survey), the online and phone questionnaires about depression symptoms ($5online survey and $10/phone survey).

**Optional Research Activities**

Passive Sensing

All participants will have a choice to opt into an additional part of this project, which involves the collection of data through a “passive sensing” application. Passive sensing describes the way someone uses their personal devices (i.e. smartphone). If you decide to take part in this part of the study, sensors that may be tracked on your phone are detailed below:

- Applications: Captures the applications being used on the smartphone.
- Communication: Captures the number of incoming/outgoing calls and SMS messages, without recording the content of these calls or messages.
- Locations: Captures your location.
- Screen usage: Captures when you lock/unlock your phone and use the screen, including time and duration of phone use. This sensor does not capture what is on the screen at any time.
- Screen interaction: Captures when you use the keyboard, without recording what is typed. This sensor also captures when you click and scroll on your smartphone.
- Battery level: Captures your device’s battery level.

We would like to collect passive sensing data for the first 9 months of the study. You will have the option to select precisely which smartphone sensors you want to activate and share with the research team and which ones you do not want to activate and share. You will also be able to select duration and timing of sensing (by turning the application on and off). Someone from the research team will show you how to do this. You will have the option to withdraw from this aspect of the study at any point, in which case the application would be uninstalled from your smartphone and no further data would be collected. The data collected through this passive sensing app will be compared to the other information collected, such as mood and wellbeing, will help researchers understand the relationship between these activity patterns and symptoms of ill-health.

Health Data Linkage

To analyse the cost-effectiveness of Rebound, we would like your permission to retrieve health data through the relevant data linkage agency in your State of residency. This will include, but is not limited to, the total number of times you have been admitted to hospital over the 18 month participation period.

Medicare and PBS

We will also ask you to fill out a separate consent for the use of your Medicare and Pharmaceutical Benefits Scheme (PBS) data. Medicare collects information on your doctor visits and lab tests while the PBS collects information on the prescription medications you have filled at pharmacies. This information helps us understand costs that may be different due to the study group to which you are assigned. This separate consent is sent securely to the Department of Human Services who holds this information confidentially. At the end of the study, your Medicare and PBS data will be linked to your other study information for analysis.

Future Research

Finally, we would like your permission to use the data we collect for this study (excluding MBS and PBS data) for future research projects that are closely related to this project but are not described here. This might include a student Honours, Masters or PhD project where the student wishes to look more closely at a specific part of the Rebound study. By giving us permission to do so, you will help us to maximize the outcome of our research effort. If you agree, we will not recontact you about the future project your data is used for. Any project using the data will have been approved by a Human Research Ethics Committee (HREC). The data will only be used in a coded way and researchers will not have access to your personalised data.

**Optional Feedback Sessions**

Meet ups

All participants allocated to group 1 maybe invited by online moderators or peer workers to meet other Rebound users at meet-ups. The purpose of these meet-ups is for Rebound participants to meet the Rebound designers and other participants as well as to share your views, experiences and suggestions using the website. Your attendance at the Rebound meet-ups will be entirely optional and will not affect in any way your ability to access the Rebound program. The Rebound meet-ups will take place at a suitable time for participants via video conferencing service. At some of these meet-ups you will be asked to participate in a focus group lasting approximately 30 minutes where the group will be asked about their experience of using Rebound. These sessions will be audio-recorded if you give your permission. During the Rebound meet-ups participants will also be asked to complete a brief questionnaire about their experience using Rebound.

**4 Other relevant information about the research project**

We plan to involve approximately 255 young people in this study. There are two groups involved in the study: Group 1 and Group 2. Both groups will have full access to all of their usual treatments and all of the appropriate treatments that are available in the community after your treatment ends.

**5 Do I have to take part in this research project?**

Participation in any research project is voluntary. If you do not wish to take part, you do not have to. If you decide to take part and later change your mind, you are free to withdraw from the project at any stage. If you do decide to take part, you will be given this Participant Information and Consent Form to sign and you will be given a copy to keep.

Your decision whether to take part or not to take part, or to take part and then withdraw, will not impact your routine treatment, your relationship with those treating you or your relationship with your treating service.

**6 What are the alternatives to participation?**

You do not have to take part in this research project to continue to receive treatment. Apart from Rebound there are other online programs currently available for mental health concerns (such as *Moodgym*) though they do not involve online social networking. You can discuss these options with your treating team. You can also discuss the options with your doctor.

**7 What are the possible benefits of taking part?**

We cannot guarantee or promise that you will receive any benefits from this research; however, possible benefits from participating in Rebound may include increased social functioning (such as increased confidence with friends) and reduced risk of experiencing a relapse of depression. In addition, by participating in this study, regardless of which group you are in, you will be assisting the researchers to find out if Rebound can assist young people in their recovery from depression.

**8 What are the possible risks and disadvantages of taking part?**

Counselling can sometimes have unintended side effects and online therapy has some specific risks. Rebound has been designed to be a private social networking site, however, it is important to understand that no website is completely “hacker proof” so there is an extremely small chance that the privacy of Rebound users could be put at risk. Other risks to privacy could include Rebound users passing on details of other Rebound users, for example via the Internet. For these reasons we are encouraging all participants who are allocated to Rebound to think about whether they use their real name or an alternative name and to keep their name confidential so that only the Rebound moderators are aware of their personal details.

It is also possible that Rebound users might communicate things in the social network that may upset others. For this reason we have set up a “report button” in Rebound so that any user can let the moderators know about anything offensive or upsetting that has been posted. If you become concerned about how Rebound is being used by other users you can also contact us by phone to report your concerns or provide feedback via Rebound.

Whilst all care will be taken to maintain privacy and confidentiality of information shared at the Rebound workshops, you may experience embarrassment if one of the group members were to repeat things said in a confidential group meeting.

You should also be aware of your personal safety when interacting with other people in an online community. For your safety we recommend that all users of the Rebound program follow cybersmart safety precautions. The Rebound website will contain a link to cybersmart information.

If you become upset or distressed as a result of your participation in the research, the researcher is able to arrange for counselling or other appropriate support. The Rebound website will provide telephone numbers that you can call for personal assistance if you become distressed. Any counselling or support will be provided by staff who are not members of the research team, for example your treating clinician or, after you have been discharged from the service, another mental health practitioner in the community. In addition, you can suspend or end your participation in the research at any time.

If you are allocated to Rebound and you become unwell during the research project, the research team may speak with you about taking a break from your involvement with Rebound until your health has improved. The team may also discuss this with your treating doctor or other health professional.

This research project involves the collection of information about your use of drugs. The questionnaire may reveal that you have previously used illegal drugs. That information will be stored in a re-identifiable (or coded) format. In the event that the research team is required to disclose that information, it may be used against you in legal proceedings or otherwise.

Because Rebound is unlike any other therapy programs, there may be additional risks that the researchers do not expect or do not know about. Tell a member of the research team immediately about any problems you experience as a result of participating in this research project.

**9 What if new information arises during this research project?**

Sometimes during the course of a research project, new information becomes available about the treatment that is being studied. If this happens, the research team will tell you about it and discuss with you whether you want to continue in the research project. If you decide to continue in the research project, you will be asked to sign an updated consent form.

Also, on receiving new information, the research team might consider it to be in your best interests to withdraw you from the research project. If this happens, he/ she will explain the reasons.

**10 Can I have other treatments during this research project?**

Whilst you are participating in this research project, you may continue to use any treatments or supports you have been accessing for any condition or for other reasons.

**11 Withdrawal from the study**

If you do consent to participate, you may withdraw at any time. You can withdraw either from the whole study, or only from the part of the study relating to your MBS and/or PBS claims information. If you decide to withdraw from either the whole research study or only the MBS and/or PBS part of the study, please notify a member of the research team. You will need to complete and sign the ‘Participant Withdrawal of Consent Form’ which is provided at the end of this document. This form should be completed and supplied to the research team. This notice will allow that person or the research supervisor to discuss any health risks or special requirements linked to withdrawing.

If you do withdraw your consent during the research project, the study coordinator and relevant study staff will not collect additional personal information from you, although personal information already collected will be retained to ensure that the results of the research project can be measured properly and to comply with law. You should be aware that data collected by the research team up to the time you withdraw will form part of the research project results. If you do not want this to happen, you should not join the research project.

**12 Could this research project be stopped unexpectedly?**

This research project may be stopped unexpectedly for a variety of reasons. These may include reasons such as:

- Unacceptable effects of Rebound
- Rebound being shown not to be effective
- Decisions made by the investigators or local regulatory/health authorities.

**13 What happens when the research project ends?**

If you are allocated to Rebound it will be available to you until the study ends (estimated to be July 2022). You will have ongoing access to all of the currently available treatments in the community. We expect that the research project will take a total of 4 years to complete, ending in the second half of 2022. If you wish to receive information about the findings of the project, please indicate this to the research team and a summary of the study results can be provided to you at the conclusion of the project. Please note that we are not able to provide you with individual research results.

**Part 2 How is the research project being conducted?**

**14 Storage, retention and destruction of your information**

By signing the consent form you consent to the study research assistant and relevant research staff collecting and using personal information about you for the research project. Any information obtained in connection with this research project that can identify you will remain confidential. Information collected by the research assistant will be stored in a secure computer file and in a locked filing cabinet at Orygen National in a non-identifiable format for a period of at least 25 years after the final publication arising from the study but may be kept indefinitely.

If you are allocated to the Rebound group your deidentified data will be linked to your Rebound account and will be accessible to the online therapist. This may help the online therapist better understand how you are doing and help to guide the treatment you are offered.

Confidentiality

We aim to keep all the information that we collect in the assessments strictly confidential. However, there are some exceptions to this: 1) information from the assessments may be communicated with your treating doctor/case manager/clinician to ensure that you receive the best care possible; 2) if we are concerned about risk to you or to someone else, we may need to discuss this with your treating doctor/case manager/clinician at the service where you receive treatment; 3) if as a result of the information you disclose in the interview relating to past trauma or abuse, we believe that someone else may be at risk. In some cases, we may contact the Department of Health and Human Services about risk to children under the age of 17 years. Mandatory reporting laws require us to report to Department of Health and Human Services any suspected cases of child abuse and neglect (Victorian Crimes Act 1958 Sect. 327). In cases where abuse is reported, information gathered by researchers is passed on to your clinical team and the appropriate clinical procedures normally used within your clinical service are implemented. This may involve reporting abuse to Department of Health and Human Services or other support services. We will try to the best of our ability to discuss this with you first.

Rebound Website

The Rebound website will be stored on an Amazon Web Services web server. The Rebound website collects usage statistics where the researchers will be able to track, if you are allocated to Rebound, which aspects of the program you have used and how often you have logged on.

Cloud Storage

Your study data (excluding MBS/PBS data) will be stored in the Cloud. “In the Cloud” refers to servers in a data centre that are managed by a third party and accessible through the Internet. When storing your study data, we will replace your name with a unique code on all your study data. The coded data will be encrypted and stored on a secure Cloud server to prevent improper access.

SEMA

Responses to questions asked via the SEMA application will be stored in a completely de-identified format on the server of the Melbourne eResearch Group (MeG) at The University of Melbourne.

If you are allocated to the Rebound group, your deidentified data will be linked to your Rebound account and will be accessible to the online therapist. This may help the online therapist better understand how you are doing and help to guide the treatment you are offered.

Passive Sensing

If you opt into the passive sensing part of this research project, data collected via the passive sensing application on your smartphone will periodically synchronise its sensing data with a central database stored on the Melbourne School of Engineering IT servers. All passive sensing data will be encrypted to ensure participants safety and confidentiality.

Medicare and PBS

Your MBS/PBS data will be retrieved from DHS at the end of the study and will be provided in a non-identifiable format. Data held on removable storage media (such as a USB stick or CD/DVD) will be stored in a locked filing cabinet initially at Deakin University then at Orygen National. Data stored electronically will be stored on the Deakin University server. All data will be password protected and the only people who will have access to this non-identifiable data and password will be the individual/s responsible for the conduct of the analysis of this data.

Your MBS/PBS data will be safely destroyed in accordance with the ‘DHS ICT Asset and Media Sanitisation Cyber Security Branch Policy and Standard’ after 7 years from the publication of the projects’ final report or after 10 years from the date of supply, whichever is sooner. A record of this will be kept in the study trial master file (TMF) which is held at Orygen National.

Audio Recording

Any audio recordings and transcriptions of the Rebound meet-ups, focus groups or user experience interview will be stored in a secure computer file at Orygen National.

Data Sharing

Your information will only be used for the purpose of this research project and it will only be disclosed with your permission, except as required by law. At times, a participant may choose to participate in more than one research project at Orygen National, OYH or headspace. In such cases, we ask that the data collected (excluding MBS/PBS data) as part of one research project may be shared with other research projects conducted at Orygen National, OYH or headspace, provided that you have consented to participate in each one individually. As many of the same measures are used across different projects, the purpose of sharing data is to prevent unnecessary repetition of assessments and ensure that participation in both projects is as simple as possible for the participant. Data collected will not be shared between current research projects unless you have signed a formal written consent form for each respective project.

Health Records

Information about you may be obtained from your treating team and from your health records held at your treating service and other health services after your treatment ends for the purpose of this research. Information about your participation in this research project will be recorded in your health records at (site). The research team may also share information gained during this research project with your treatment team. By signing the consent form you agree to the study team accessing your health records if they are relevant to your participation in this research project.

Your health records and any information obtained during the research project are subject to inspection (for the purpose of verifying the procedures and the data) by the relevant authorities and authorised representatives of Orygen National, The Melbourne Health Human Research Ethics Committee, or as required by law. By signing the Consent Form, you authorise release of, or access to, this confidential information to the relevant study personnel and regulatory authorities as noted above.

Publication

It is anticipated that the results of this research project will be published and/or presented in a variety of forums. In any publication and/or presentation, information will be provided in such a way that you cannot be identified, except with your permission. Information that is published from this study will only include summary information that describes the whole group of participants in this study and not to any individual participant.

Freedom of Information

In accordance with relevant Australian and Victorian privacy and other relevant laws, you have the right to request access to your information collected and stored by the research team. You also have the right to request that any information with which you disagree be corrected. Please contact the study team member named at the end of this document if you would like to access your information.

Disclosure of Drug Use

Any information obtained for the purpose of this research project and for future research described in Section 3 that can identify you will be treated as confidential and securely stored. It will be disclosed only with your permission, or as required by law. This research project involves the collection of information about your use of drugs. That information will be stored in a re-identifiable (or coded) format. In the event that the research team is required to disclose that information, it may be used against you in legal proceedings or otherwise.

Data Repository

Your data (excluding MBS and PBS data) may be included in a data repository that allows researchers to collect and share coded information with each other.  Coded information means that all personal information about research participants such as name, address, and phone number is removed and replaced with a code number. With an easier way to share, researchers hope to learn new and important things more quickly than before.

This means researchers may send coded information about your study data to a data repository.  Researchers who would like to access your data for research purposes would need to request this of the database administrators to obtain access to your study data for research purposes only. These database administrators would know how to protect health and science information and will look at every request carefully to minimize risks to your privacy.  It is not known at this stage whether or not the data repository will include national or international members, so by consenting to your data being used for future research, you may be consenting to transfer of your data overseas.

**15 Complaints and compensation**

The person you may need to contact will depend on the nature of your query.

If you want any further information concerning this project or if you have any medical problems which may be related to your involvement in the project (for example, any side effects), you can contact the following people:

**Clinical contact person**

| Name | Professor Mario Alvarez-Jimenez |
| --- | --- |
| Position | Director Orygen Digital |
| Telephone | 0401772668 |
| Email | mario.alvarez@orygen.org.au |

If you or have any complaints about any aspect of the project, the way it is being conducted or any questions about being a research participant in general, then you may contact the reviewing HREC approving this research:

**Reviewing HREC approving this research and HREC Office contact details:**

| Name | Ms Jessica Turner |
| --- | --- |
| Position | Manager, Melbourne Health Human Research Ethics Committee |
| Telephone | (03) 9342 8530 |
| Email | [research@mh.org.au](mailto:research@mh.org.au) |

**Complaints contact person**

If you have any complaints about any aspect of the project then you may contact:

| Name | Insert local contact details |
| --- | --- |
| Position |  |
| Telephone |  |
| Email |  |

If you suffer any injuries or complications as a result of this research project, you should contact the study team as soon as possible and you will be assisted with arranging appropriate medical treatment. You can receive any medical treatment required to treat the injury or complication, free of charge, as a public patient in any Australian public hospital.

If you suffer an injury as a result of your participation in this research project you may be able to seek compensation through the courts. If you suffer any distress or psychological injury as a result of this research project, you should contact the research team as soon as possible. You will be assisted with arranging appropriate treatment and support.

**16 Who is organising and funding the research?**

This research project is funded by The National Health and Medical Research Council.

Orygen National, , The Australian Catholic University and the Department of Computing and Information Systems at The University of Melbourne may benefit financially from this research project if, for example, the Rebound website is commercialised.

You will not benefit financially from your involvement in this research project.

In addition, if knowledge acquired through this research leads to discoveries that are of commercial value to Orygen National, The Australian Catholic University and the Department of Computing and Information Systems at The University of Melbourne, there will be no financial benefit to you or your family from these discoveries.

No member of the research team will receive a personal financial benefit from your involvement in this research project (other than their ordinary wages).

**17 Who has reviewed the research project?**

All research in Australia involving humans is reviewed by an independent group of people called a Human Research Ethics Committee (HREC). The ethical aspects of this research project have been approved by the HREC of Melbourne Health.

This project will be carried out according to the National Statement on Ethical Conduct in Human Research (2007). This statement has been developed to protect the interests of people who agree to participate in human research studies.

**Consent Form -** *Adult providing own consent*

| **Title** | Preventing relapse of major depressive disorder in youth: RCT of a novel mindfulness-based cognitive online social therapy |
| --- | --- |
| **Short Title** | Rebound |
| **Protocol Number** | 2018.217 |
| **Project Sponsor** | Orygen National |
| **Coordinating Principal Investigator** | Professor Mario Alvarez |
| **Principal Investigator** | (Insert site PI) |
| **Study Coordinator** | Mrs Daniela Cagliarini |
| **Location** | - (Insert location) |

**Consent Agreement**

I have read the Participant Information Sheet or someone has read it to me in a language that I understand.

I understand the purposes, procedures and risks of the research described in the project.

I consent to the study team accessing my health record and sharing information with my treating team as relevant to the research project.

I give permission for my doctors, other health professionals, hospitals or laboratories to be contacted by the researchers and to release information to Orygen National, concerning my disease and treatment for the purposes of this project. I understand that such information will remain confidential.

I have had an opportunity to ask questions and I am satisfied with the answers I have received.

I freely agree to participate in this research project as described and understand that I am free to withdraw at any time during the study without affecting my future health care.

I consent to my data being shared between research projects conducted at Orygen National, OYH and headspace provided that I have signed a formal written consent form to participate in each project. I understand that I can withdraw my consent for this to occur at any time without affecting my participation in either study or my future health care.

I understand that I will be given a signed copy of this document to keep.

In addition, please indicate whether you give consent for the following **optional** activities:

I agree to participate in the passive sensing part of this study.

Yes □ No □ Participant Initials:_______________ Date:________________

I agree to the collection of my health data through the data linkage agency in my state of residency.

Yes □ No □ Participant Initials:_______________ Date:________________

I agree to the collection of my Medicare/PBS data.

Yes □ No □ Participant Initials:_______________ Date:________________

I agree to my data being used for future research.

Yes □ No □ Participant Initials:_______________ Date:________________

I agree to audio-recording and transcription of any feedback session I attend as part of my involvement in this study.

Yes □ No □ Participant Initials:_______________ Date:________________

I agree to my data being included in a data repository.

Yes □ No □ Participant Initials:_______________ Date:________________

**Declaration by Participant**

| Name of Participant (please print) _________________________________________________  Signature _______________________________ Date _______________________________ |
| --- |

| Witness to the informed consent process  Name (please print) __________________________________________________________  Signature _______________________________ Date ______________________________  * Witness is not to be the investigator, a member of the study team or their delegate. In the event that an interpreter is used, the interpreter may not act as a witness to the consent process. Witness must be 18 years or older. |
| --- |

**Declaration by Study Doctor/Senior Researcher^†^**

I have given a verbal explanation of the research project, its procedures and risks and I believe that the participant has understood that explanation.

|  | | | | | | |
| --- | --- | --- | --- | --- | --- | --- |
|  | Name of Study Doctor/  Senior Researcher^†^ (please print) | |  | | |  |
|  | | | | | |  |
|  | Signature |  | | Date |  |  |
|  | | | | | | |

^†^ A senior member of the research team must provide the explanation of, and information concerning, the research project.

Note: All parties signing the consent section must date their own signature.

**Form for Withdrawal of Participation -** *Adult providing own consent*

| **Title** | Preventing relapse of major depressive disorder in youth: RCT of a novel mindfulness-based cognitive online social therapy |
| --- | --- |
| **Short Title** | Rebound |
| **Protocol Number** | 2018.217 |
| **Project Sponsor** | Orygen National |
| **Coordinating Principal Investigator** | Professor Mario Alvarez |
| **Principal Investigator** | (Insert Site PI) |
| **Study Coordinator** | Mrs Daniela Cagliarini |
| **Location** | - (Insert trial site) |

**Declaration by Participant**

I wish to withdraw from participation in the above research project and understand that such withdrawal will not affect my routine treatment, my relationship with those treating me or my relationship withmy treating service.

Please tick one of the following boxes:

I wish to withdraw my participation and allow researchers to use all my information provided up until the point of withdrawal.

I wish to withdraw my participation and have all my information destroyed from the whole study, where possible, and have no further participation.

I wish to withdraw my participation and have all my Medicare Benefits Schedule (MBS) and/or Pharmaceutical Benefits Scheme (PBS) claims destroyed from the study where possible.

I wish to withdraw my participation but allow my MBS and/or PBS claims collected up to the withdrawal date to continue to be used in the study.

I understand that:

1. no further information about me will be collected for the study from the withdrawal date;
2. my information that has already been collected, and analysed and/or included in a publication, may not be able to be withdrawn or destroyed; and
3. my withdrawal from the study will not affect my access to Health Services or government benefits, and where relevant, will have no bearing on the medical care I receive.

|  | | | | | | |
| --- | --- | --- | --- | --- | --- | --- |
|  | Name of Participant (please print) | |  |  |  |  |
|  | | | | | | |
|  | Signature |  | | Date |  |  |
|  | | | | | | |

*In the event that the participant’s decision to withdraw is communicated verbally, the Study Doctor/Senior Researcher will need to provide a description of the circumstances below.*

|  |
| --- |

**Declaration by Study Doctor/Senior Researcher^†^**

I have given a verbal explanation of the implications of withdrawal from the research project and I believe that the participant has understood that explanation.

|  | | | | | | |
| --- | --- | --- | --- | --- | --- | --- |
|  | Name of Study Doctor/  Senior Researcher^†^ (please print) | |  | | |  |
|  | | | | | |  |
|  | Signature |  | | Date |  |  |
|  | | | | | | |

^†^ A senior member of the research team must provide the explanation of and information concerning withdrawal from the research project.

Note: All parties signing the consent section must date their own signature.

This form should be forwarded by email to: daniela.cagliarini@orygen.org.au.

Alternatively, forms can be posted to:

Daniela Cagliarini

Orygen National

35 Poplar Rd, Parkville 3052.
