# Supplementary material for: A mindfulness-based, cognitive, social, digital relapse-prevention intervention for youth with depression in Australia: study protocol for a randomised controlled trial of Rebound
Source: BMJ Open. 2024 Nov 27;14(11):e088695. doi: 10.1136/bmjopen-2024-088695 (PMC11603739; doi:10.1136/bmjopen-2024-088695)
Supplement: Supplementary file 2 [file bmjopen-2024-088695.reviewer_comments.pdf]

PEER REVIEW HISTORY

BMJ Open publishes all reviews undertaken for accepted manuscripts. Reviewers are asked to complete a checklist review form and are provided with free text boxes to elaborate on their assessment. These free text comments are reproduced below.

ARTICLE DETAILS

Title (Provisional)

Study protocol for a RCT of Rebound: a mindfulness-based digital relapse prevention intervention for youth with depression in Australia

Authors

Mangelsdorf, Shaminka N.; Cagliarini, Daniela; Lee, Yong Yi; Mihalopoulos, Cathrine; Liu, Virginia; Valentine, Lee; Bendall, Sarah; Koval, Peter; D'Alfonso, Simon; Davey, Christopher; Russon, Penni; Phillips, Jess; Gonzalez-Blanch, Cesar; Pawsey, Brendan; Ryan, Richard M; Parker, Alexandra; Hetrick, Sarah; Rice, Simon; Lederman, Reeva; Herrman, Helen; Murray, Greg; Gleeson, John; Alvarez-Jimenez , Mario

This article was not externally reviewed at BMJ Open. Protocols that have been independently assessed prior to submission to BMJ Open are usually fast-tracked to publication on the grounds that further substantial changes will not be possible. This independent assessment will usually be external, independent review for both a substantial grant award from a non-commercial or government funder and ethics approval.
